# Antibacterial Properties of Polyurethane Foams Additivated with Terpenes from a Bio-Based Polyol

**DOI:** 10.3390/molecules28041966

**Published:** 2023-02-18

**Authors:** Simona Tomaselli, Fabio Bertini, Angelica Cifarelli, Adriano Vignali, Laura Ragona, Simona Losio

**Affiliations:** SCITEC-CNR Institute for Chemical Sciences and Technologies “G. Natta” National Research Council, Via A. Corti 12, 20133 Milan, Italy

**Keywords:** polyurethane foams, terpenes, antibacterial properties, NMR

## Abstract

Water-blown polyurethane (PU) foams were prepared by bio-polyols from epoxidized linseed oils and caprylic acid in combination with toluene diisocianate (TDI). A series of terpenes (menthol, geraniol, terpineol, and borneol), natural compounds with recognized antibacterial properties, were included in the starting formulations to confer bactericidal properties to the final material. Foams additivated with Irgasan^®^, a broad-spectrum antimicrobial molecule, were prepared as reference. The bactericidal activity of foams against planktonic and sessile *E. coli* (ATCC 11229) and *S. aureus* (ATCC 6538) was evaluated following a modified AATCC 100-2012 static method. Menthol-additivated foams showed broad-spectrum antibacterial activity, reducing Gram+ and Gram− viability by more than 60%. Foams prepared with borneol and terpineol showed selective antibacterial activity against *E. coli* and *S. aureus*, respectively. NMR analysis of foams leaking in water supported a bactericidal mechanism mediated by contact killing rather than molecule release. The results represent the proof of concept of the possibility to develop bio-based PU foams with intrinsic bactericidal properties through a simple and innovative synthetic approach.

## 1. Introduction

Polyurethanes (PU) are currently among the most extensively used polymers in the world, with an estimated production volume of 29 million tons by 2029 [1]. Thanks to the wide range of their achievable properties, they have been used in various applications such as adhesives, coatings, foams, and packages [2,3,4,5].

Conventional PUs are derived from isocyanate and hydroxy compounds and/or amines [6], and their properties and processability are based on the chemical nature of the polyols and chain extenders, reaction parameters as well as the use of catalysts [7]. In the last two decades, an increasing interest in the preparation of polymers from renewable resources, i.e., bio-based polymers derived from living organisms such as plants, trees and algae, has been observed due to declining non-renewable feedstock [8,9,10]. Implementing the use of natural products for the replacement of petrochemical-based monomers has the potential to decrease the dependence on fossil fuels and increase the number of material applications of more renewable resources. One way of expanding the content of natural products in polymeric materials is through the incorporation of bio-based materials and their derivatives as monomers [11]. Bio-derived materials, such as vegetable oils, constitute a rich source of precursors for the synthesis of polyols, which can be considered for the production of PU with a higher green carbon content. The main component of vegetable oil is triglyceride, which consists of an ester of glycerol and three long-chain fatty acids with varying composition depending on the source of the oil [12,13]. Alkene groups are the most important reactive sites used to obtain monomers or to generate polymers. In particular, fatty acids can be converted into polyols through different production methods. Among them, epoxidation reactions followed by ring-opening of the oxirane group have been widely investigated for the preparation of bio-based polyols [14,15,16,17]. Along this line, some of us recently reported an environmentally friendly, solvent-free method for the preparation of bio-polyols from epoxidized soybean and linseed oils and caprylic acid or 3-phenyl butyric acid in the presence of triethylamine as catalyst [18]. The obtained bio-polyols, in a mixture with polypropylene glycol (PPG), were used with Tolonate^TM^, an aliphatic bio-based diisocyanate, to prepare flexible polyurethane foams [18]. Despite the reduced carbon footprint given by the diisocyanate, the presence of the bio-based polyols led to slightly worse mechanical properties with respect to that of a reference foam obtained in the presence of the alone PPG, especially when bio-polyols from 3-phenyl butyric acid were used. These results prompted us to go further with the development of new foams and a series of novel PU foams have been prepared in the presence of bio-polyols from caprylic acid with the well-known commercial diisocyanate, toluene diisocianate (TDI). The obtained flexible bio-based PU are intended to be used for applications in health care, cosmetology, pharmacology or food where an aseptic environment is often required. For this reason, conferring intrinsic antibacterial properties to PU is of particular interest and avoids the usage of disinfectants, which are toxic when released in the environment.

Today, to confer antibacterial properties to polymers for everyday use products, the tendency is to employ natural, bio-based additives instead of synthetic ones. So far, a few reports on the use of natural antibacterial substances in foams have been reported in the literature [19]. Significant antibacterial activity against *E. coli* and *S. Aureus* has been described for PU rigid foams embedding clove filler [20], nutmeg compound [21], cinnamon extracts [22] or curcumin [23]. Antibacterial PU was also obtained via coating procedures with clove oil [24]. Antibacterial properties have also been shown for PU films modified by covalently linking chitosan and heparin by activating the surface with plasma and glutaraldehyde [25].

In the present work, we propose an innovative strategy to prepare foams with intrinsic antibacterial properties by incorporating terpene monomers. Terpenes are a large class of natural compounds displaying important biological functions, especially in the defense mechanism of many organisms [26]. The name “terpene” was assigned by Kekulè and derives from “turpentine”, the so-called oil distilled from pine resin [27]. Terpenes are derived from the isoprenoid pathway [26]. They are composed of isoprene units (C5), which is the basis for their classification, i.e., two isoprene units form monoterpenes (C10), three units form sesquiterpenes (C15), four units form diterpenes (C20), six units form triterpenes (C30) and eight units form carotenoids (C40) [28]. Different chemical functionalities can be observed in terpenes: alcohol (such as terpineol, menthol, borneol, carveol, and geraniol), aldehyde (citral and citronellal), phenol (thymol and carvacrol), ether (eucalyptol) and hydrocarbon (cymene, pinene and limonene) groups [28]. Terpenes display different biological activities such as antimicrobial, anticarcinogenic, and antioxidative effects. In general, the oxygenated terpenes have been reported to exhibit better antibacterial activity than the hydrocarbon congeners [29,30,31]. In detail, oxygenated monoterpenes, especially phenolic compounds [32,33,34,35], exhibit strong antimicrobial activity, mainly pronounced on whole cells. In contrast, the lower antimicrobial activity observed for hydrocarbon derivatives seems originated from their low water solubility, limiting their diffusion [36]. Ketones, aldehydes and alcohols are active too, but with levels of activity related to their functional group [26]. In addition to the use of terpenes in the fine chemical and fragrance industries, thanks to their pleasant and balsamic smell, there has been much interest in their use in polymeric materials. Currently, several industrially relevant polymerization processes are known which take advantage of widely abundant terpenes, e.g., the cationic polymerization of α-pinene for adhesives, coatings, and inks [37]. Very recently, the Meier group illustrated the research and development of nitrogen-containing polymers such polyamides, polyurethanes (and their non-isocyanate counterparts), polyureas, and epoxy resins, using terpenes and their derivatives as monomers [38]. Terpene-modified polysiloxane-based polyurethane was also developed to improve mechanical properties of waterborne polyurethane [39]. Moreover, hyper-cross-linked porous polymers loaded with L-borneol with interesting antibacterial and antipollution properties have been prepared as well [40].

In this work, we investigated whether the presence of selected terpenes in water-blown PU foam formulations confers antibacterial properties to the newly developed materials. A series of terpenes, with known bactericidal activity, has been selected (Table 1).

**Scheme 1:**
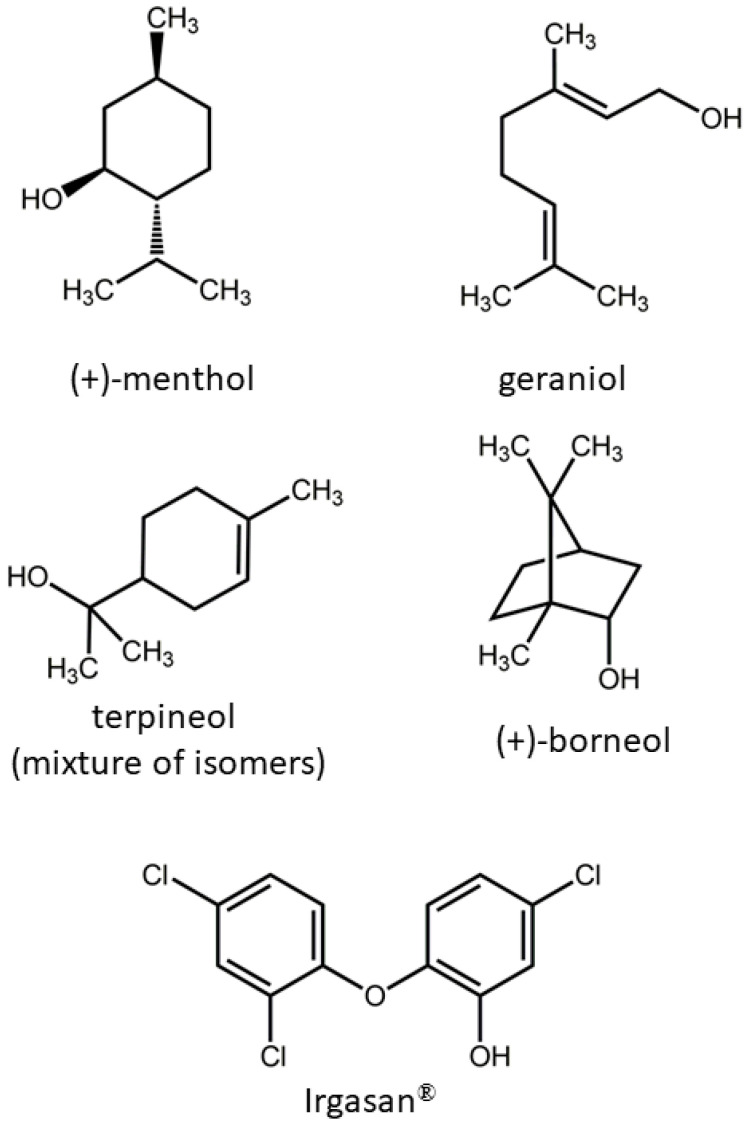
Molecular structure of terpenes used in this study and Irgasan^®^.

Several foams have been prepared from bio-polyol, obtained by epoxidized linseed oil (ELO) and caprylic acid, and TDI with different terpenes such as (+)-menthol, geraniol, terpineol (mixture of isomers), and (+)-borneol (Scheme 1). A foam prepared with Irgasan^®^ (IRG) has been prepared as well. Irgasan^®^ or 5-chloro-2-(2,4-dichlorophenoxy)phenol (Scheme 1) is a commercial broad-spectrum antibacterial agent effective against many types of bacteria and certain types of fungi [47,48]. IRG has been selected as reference for the optimization of the synthesis process and antibacterial tests in light of its high efficacy and widespread use in many consumer products, including cosmetics, kitchen utensils, clothing textiles, electronics, plastics, and toys.

The antibacterial properties of the prepared foams against both Gram− and Gram+ bacteria strains have been fully characterized through viability tests. Foam morphology, thermal and mechanical properties were evaluated by SEM, TGA and mechanical compressive investigations. NMR approaches have been employed to detect any eventual leaking of components from the newly prepared foams over time.

To the best of our knowledge, the described bio-based foams are the first example of PU foams in which the bactericidal molecule of natural origins has been introduced into the formulation, conferring intrinsic antibacterial properties to the final material, without significant active molecule release in water. 

## 2. Results and Discussion

### 2.1. Preparation and Characterization of Bio-Polyurethane Foams

A series of bio-polyurethane foams was prepared by the reaction of commercial aromatic di-isocyanates such as TDI with a bio-polyol (from now on BPO) from commercial epoxidized linseed oil as renewable-based polyfunctional building blocks [18]. The BPO has been synthetized by a solvent-free method already reported by some of the authors, where the ring-opening reaction of the oxirane group of ELO with caprylic acid occurs in the presence of triethylamine (TEA) to facilitate the ring opening of the internal epoxy groups of ELO. The reaction has been conducted at 150 °C for 4 h. At the end of the reaction, each reacted epoxide group is converted in an OH group, an ester linkage is formed between the main chain of the polyol and the organic acid as a result of the nucleophilic attack on the epoxide ring. The commercial epoxidized linseed oil was previously characterized, showing 6.40 epoxy groups per triglyceride.

The properties of the BPO are summarized in Table 2. 

The final properties of the bio-polyol, such as low OH functionalities (between 2 and 3) or molecular weights up to 2000 g/mol, are key issues for the preparation of flexible PU foams. 

The formulation of bio-based polyurethane foam is reported in Table 3. A selected ratio PPG:bio-polyol, equal to 1:1, was chosen, and a chemical blowing agent (distilled water) was used (1.5 wt%). As a blowing catalyst, DABCO was tested for all the formulations and a constant amount of dibutyltin dilaurate (DBT) (0.1 pphp) was fixed. An amount of glycerol, 6 wt/wt%, was used to enhance the bio-polyol miscibility with water.

To the starting formulation (PUC), various terpenes such as (+)-menthol, geraniol, α-terpineol as a mixture of isomers, (+)-borneol, and Irgasan^®^ were added before the foaming (Table 4). A wide range of concentrations were investigated (between 2 and 10 wt/wt%); however, for each terpene, a concentration limit has been selected to preserve the dimensional stability (shrinkage up to 2%) and the foam properties. Geraniol and α-terpineol, showing a higher reported MIC, were added at a higher wt%.

In general, the density of water-blown PU foams depends on the amount of CO_2_ produced, which further depends on the amount of water added. For this reason, a low amount of water was used as a blowing agent (1.5 wt/wt%), to better control the distribution of the additives inside low-density structures. PUC shows an apparent density of 120 Kg/m^3^ while the foams containing all the terpenes show a density ranging from 85 to 150 Kg/m^3^. It is worth noting that each additive, depending on its concentration, impacts on the final density of the foam, with density decreasing at a higher amount of the same additive (Table 4).

**Figure 1:**
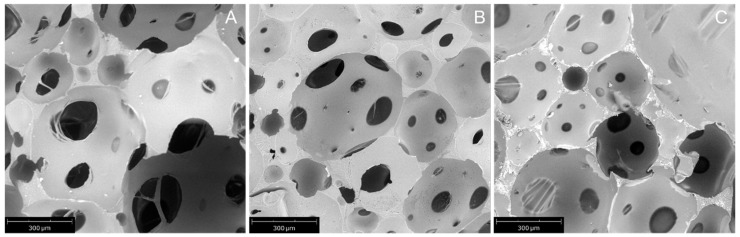
SEM micrographs (magnification 225×) of PUC (**A**), 1_menthol (**B**) and 1_borneol (**C**) foams.

Foam morphology and the thermal and mechanical properties were evaluated by SEM, TGA and mechanical compressive investigations. Hereafter, the characterization of the reference foam, PUC, and two selected foams showing antibacterial activity, i.e., 1_menthol and 1_borneol (*vide infra*), is reported. In Figure 1, SEM micrographs of selected foams are depicted, showing the cross-section surface of the samples. 

All foams have an open-cell structure, with well-developed cavities and interconnected pores. The structure of foams presents an approximately spherical shape of pores and cavities, with a relatively homogeneous distribution and diameters ranging 50–250 µm and 300–600 µm, respectively, determined according to a previous work [49]. Moreover, few closed pores were observed. These pores, along with the presence of smaller ones, could justify the higher values of mechanical properties compared to the foam based on bio-polyols investigated in our previous work [18]. 

**Figure 2:**
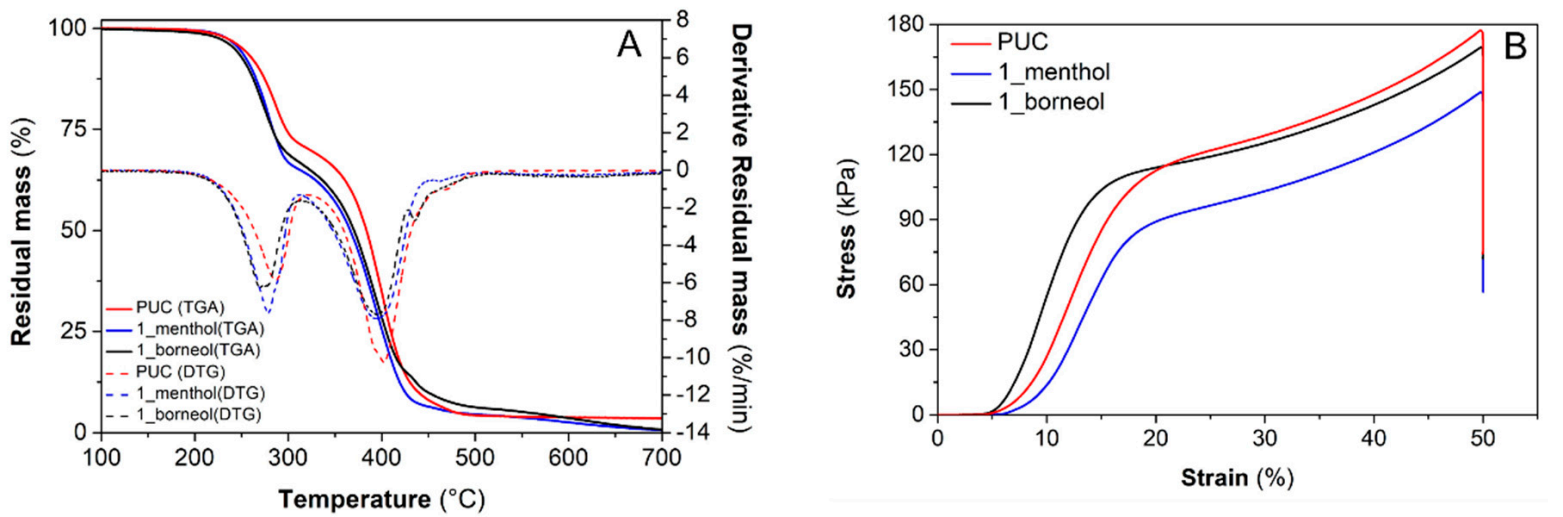
TGA-DTG thermograms (**A**) and stress–strain curves (**B**) of PUC, 1_menthol and 1_borneol foams.

TGA and derivative thermogravimetry (DTG) curves of PUC, 1_menthol and 1_borneol foams are depicted in Figure 2A. Thermograms show a very similar thermal behavior with a two-step thermal degradation under nitrogen atmosphere. The first mass loss step, recorded between 200 and 320 °C, is associated with approximately 30% of the mass loss as a result of the dissociation of the urethane bonds, whereas the second degradation step, observed at higher temperatures (320–500 °C), is ascribed to chain scission in the bio-polyol structures [18]. In detail, the two degradation steps exhibited temperatures of maximum rate of mass loss, determined corresponding to DTG peaks, at approximately 280 and 395 °C, respectively. Moreover, the sample showed good thermal stability, with a starting decomposition temperature, evaluated at an initial 5% mass loss, ranging from 240 to 250 °C.

The compression stress–strain curves of selected samples are shown in Figure 2B. The antibacterial foams present mechanical properties analogous to the non-additivated reference sample (PUC). Firstly, the curves exhibit an elastic linear stage up to 15% of strain, in which the stress strongly rises with strain as result of cell deformation. Subsequently, yield and progressive increase in stress, related to a partial densification, were observed. In particular, the selected foams are relatively rigid with a Young’s modulus and compression deflection value, determined as the ratio between the final load and the cross-section area of the specimen, in the range of 1100–1300 kPa and 60–75 kPa, respectively (Table 5).

### 2.2. Evaluation of Bactericidal Activity against Planktonic and Sessile Bacteria

**Figure 3:**
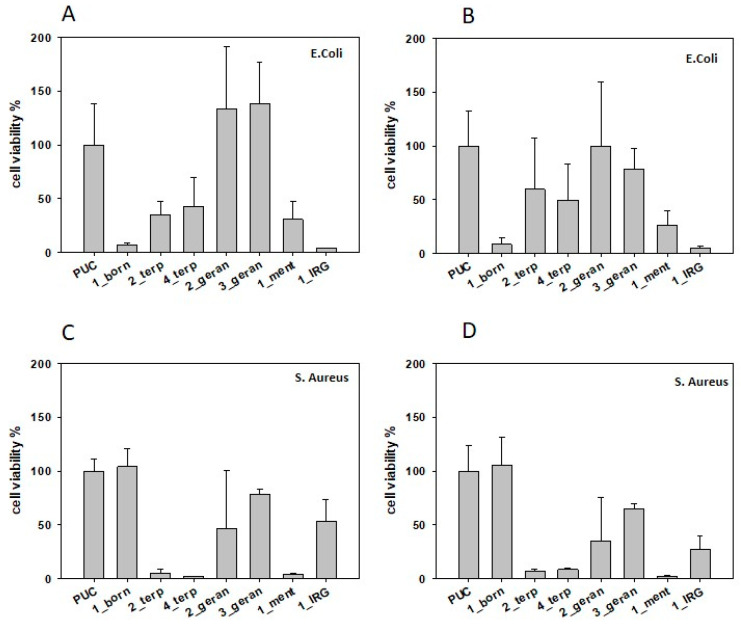
Percentage of cell viability of planktonic (**A**,**C**) and sessile (**B**,**D**) *E. Coli* and *S. Aureus* in the presence of 1_borneol, 2_terpineol, 4_terpineol, 2_geraniol, 3_geraniol, 1_menthol, and 1_irgasan additivated foams and their control devoid of additives (PUC), after 24 h contact time.

The antibacterial properties of polyurethane foams (Table 4) were tested on Gram+ and Gram– bacteria using a modified AATCC 100-2012 static method, which involves the immersion of specimens in 1 mL liquid and subsequent estimation of bacterial survival upon contact. Additive-free foam was used as reference. Foams obtained from BPO in the presence of different terpenes and Irgasan^®^ showed different antibacterial activity when tested against *E. Coli* and *S. Aureus*. Tests performed on planktonic cells showed that 1_borneol reduces viability (<10%) when tested against *E. Coli* (Figure 3A), while it is not active against *S. Aureus*. In contrast, foams with terpineol strongly reduce *S. Aureus* viability below 10% (Figure 3C), and are mildly effective against *E. Coli* even for 4_terpineol, containing a higher amount of additive. Interestingly, 1_menthol foams showed a significant reduced viability (≤20%) for both *E. Coli* and *S. Aureus* (Figure 3A,C). IRG significantly reduced cell viability after 24 h (<10%) for *E. Coli* and exhibited a mild activity against *S. Aureus*. Foams additivated with geraniol did not show any significant antibacterial activity at both tested additive wt%.

The foams were also tested for their ability to prevent biofilm formation. The percentage of cell viability is reported in Figure 3B,D. The results indicate that foams that are able to reduce planktonic cells are also able to reduce biofilm formation. It is important to underline that the antibacterial activity of these molecules was unaltered even after eight months from synthesis (data not shown).

### 2.3. Evaluation of Foam Component Leaking by NMR

To investigate whether the antibacterial properties could be due to the release of specific components from the foams, leaking effects were evaluated by NMR. Each specimen was immersed in 1 mL KPi and kept at 37 °C for 24 h, and then the 1D ^1^H NMR spectra were recorded. 

**Figure 4:**
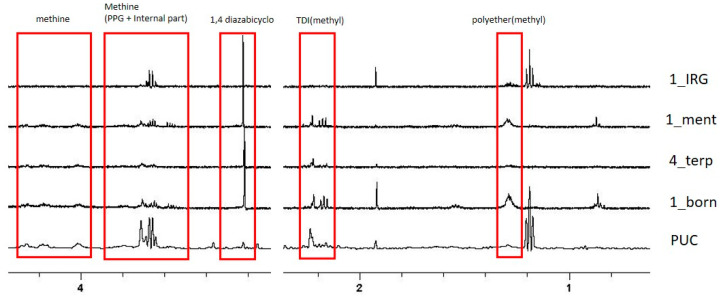
Selected regions of 1D ^1^H NMR spectra of 1_irgasan, 1_ menthol, 4_terpineol, and 1_borneol and PUC are shown. Assignment of selected peaks is reported.

Figure 4 shows selected regions of 1D ^1^H NMR spectra obtained for samples with the highest antibacterial activity (1_borneol, 4_terpineol, 1_menthol and 1_irgasan) and for the control (PUC).

PUC and all foam spectra show the presence of a low concentration of residual formula components, signals of the 1,4-diazabicyclo[2.2.2]octane catalyst, used for the formulation, observed at 3.2 ppm. Spectra also show the presence of several resonances that can be ascribed to TDI isocyanate along with PPG labelled (in 1_borneol at higher concentration). All these substances are present at low concentrations, estimated in the range 6–10 µM, on the basis of the relative abundance with respect to TSP (0.1 mM), added as an internal reference to the NMR samples. It is reasonable to assume that the released components are not responsible for foam antibacterial activity as their concentrations are variable in the five samples and do not seem to correlate with the antibacterial activity deduced from viability tests. 

Signals of terpenes were not observed in any NMR spectrum, suggesting that their eventual concentration is lower than 10 μM, significantly lower than the MIC value reported in the literature (Table 1). Altogether, NMR data suggest a bactericidal action which relies on surface contact killing mechanisms, rather than on the release of toxic molecules in solution. 

The antibacterial activity of non-leaching bound additive polymers is exerted on the surface of the material via a physical interference with bacteria cells. These molecules, although covalently bound, are exposed on the material surface and exert their antimicrobial efficacy by physically disrupting the bacterial cell wall and membrane [50]. Quaternary ammonium salt, N-halamine and chitosan covalently bound polyurethanes are examples of non-leaching surface contact killing materials, whose active molecules are able to disrupt the cell wall or membrane, yielding to cell death, through different mechanisms related to the chemical nature of the additives. Quaternary ammonium salts and chitosan are positively charged; they interact with the negative charges of the cell wall, destabilizing and weakening the cytoplasmic membrane, causing a loss of cytoplasmic constituents. N-halamine directly transfers the oxidative state of halide atoms in chloramine or bromamine groups to the cell wall of the microorganism [51]. It was shown that terpenes trigger different mechanisms, leading to the loss of cellular membrane integrity [29,52], or enter the cell, causing cell death [44]. In particular, it has been demonstrated that terpineol directly disrupts the cell membrane [29], and menthol perturbates the cell surface [53], while geraniol activity is more related to its ability to penetrate into the interior of the bacterial cell and subsequently interact with intracellular sites [45]. It has widely been demonstrated that IRG primarily targets the cell membrane, damaging and destabilizing it. NMR data demonstrated that IRG intercalates within hydrophobic pockets in the lipid bilayer, perpendicularly to phospholipid molecules [48,54]. We are aware that the determinants of the antibacterial effectiveness of a bactericidal material depends on a combination of factors, related to the single additive properties and to foam features (density, porosity, etc.). However, it is interesting to note that the variable antibacterial performance of polyurethanes foams observed for the newly developed materials correlate well with the mechanism of action of the additives. Menthol, terpineol and IRG, although covalently bound, when exposed on the material surface, can directly interact and disrupt cell membranes, thus conferring bactericidal activity to foams. In contrast, geraniol, which needs to penetrate the membrane to exert its intracellular activity, was not able to confer significant bactericidal properties to the non-leaking foam. Further studies, through real-time NMR analysis of extracellular metabolites produced by bacteria grown in the presence of foams [46], are currently ongoing in our laboratories to clarify the molecular basis of the bactericidal action of the discussed terpene-additivated foams.

## 3. Materials and Methods

### 3.1. General Considerations

All bio-polyol syntheses were carried out on a double manifold Schlenk vacuum line under a nitrogen atmosphere according to the literature [18]. Epoxidized linseed oil (ELO) with approximately 6.4 epoxy groups per triglyceride and *M*_n_ equal to 1182 g/mol was kindly supplied by Hallstar (Chicago, IL, USA). 1,4-diazabicyclo[2.2.2]octane (DABCO, 98%), toluene-2,4-diisocyanate (TDI, tech. 80%, remainder 2,6-diisocyanate) glycerol (≥99%) and triethylamine (TEA, 98%) were purchased from Fisher Scientific Company (Hampton, NH, USA). Caprylic acid (CA, ≥99%) was purchased from Carlo Erba (Milan, Italy). Niax Silicone L-537 XF (Momentive, NY, USA) was kindly supplied by Eigenmann & Veronelli SpA (Milan, Italy) and used as silicone surfactant. Polypropylene glycol (PPG, Mn ~4600 g/mol from SEC analysis), all the terpenes, dibutyltin dilaurate (DBT) and Irgasan^®^ were purchased from Merck KGaA (Darmstadt, Germany). Deuterated solvent, CD_2_Cl_2_, and hexamethyldisilane (HMDS) for NMR measurements were used as received from Merck KgaA (Darmstadt, Germany).

### 3.2. Foam Formulation

The formulations of bio-based polyurethane foams are reported in Table 3. A selected ratio equal to 1:2 PPG:bio-polyol was chosen, and a chemical blowing agent (distilled water) was used (1.5 wt%). Bio-polyol was prepared according to the literature and their properties reported in Table 2 [18]. As blowing catalyst, DABCO was tested for all the formulations, and a constant amount of DBT (0.1 pphp) was fixed. An amount of glycerol, 6 wt/wt%, was used to enhance the bio-polyol miscibility with water.

Starting from the formulation of the bio-based polyurethane foams, numerous samples with different amounts of additives have been prepared (Table 4). After 15–20 min of manufacturing, the foams were mechanically crushed to open up closed cells and to avoid shrinking of the foam. Once obtained, the foams were washed with iso-propanol for 3 h and then with distilled water at 60 °C for 2 h. Then, the samples were sterilized in H_2_O/ethanol 70/30 and 80/20 wt/wt% for 2 h.

### 3.3. Characterization Techniques

The hydroxyl number (OH number, mg KOH/g) of the polyol was determined by the imidazole-catalyzed pyromellitic dianhydride method, according to ASTM D 4274-99. The acid value was determined according to the IUPAC 2.201 standard using the indicator method.

The viscosity of the polyol was determined by using a TA Instruments (New Castle, DE, USA) AR 2000 rotational rheometer at 23 °C with a flow test in the shear rate range 0.01–1000 s^−1^. The rheometer was equipped with a plate and steel cone (diameter of 20 mm, angle of 0.5° and truncation gap of 16 µm).

Size-exclusion chromatography (SEC) analysis was performed on a Waters (Mil-ford, MA, USA) GPCV2000 system, using THF as the mobile phase, at 35 °C with a 0.6 mL/min flow. The sample concentration was set at 2 mg/mL and the injection volume at 150 µL. The SEC system was calibrated using polystyrene standards.

Thermogravimetric analysis (TGA) was carried out on a PerkinElmer (Waltham, MA, USA) TGA 7 instrument from 50 to 750 °C at a heating rate of 10 °C/min under nitrogen atmosphere.

The mechanical behavior of the foams was evaluated through compression force deflection tests performed by a Zwick-Roell (Ulm, Germany) Z010 mechanical testing machine with a load cell of 2.5 kN. Cylindrical specimens (height of 20 mm and diameter of 45 mm) were initially pre-compressed twice to 75% of their initial height at a cross-head speed of 250 mm/min. After a rest of 6 min, the specimens were compressed up to 50% of their height at 50 mm/min and the final load after 60 s was measured. Reported data were averaged from at least three tests per sample.

The morphology of foams was investigated using a Thermo Fisher Scientific (Waltham, MA, USA) Phenom XL G2 scanning electron microscope (SEM) operating at 10 kV. Before the observations, the samples were cut with a blade from the middle of the foams.

### 3.4. Antibacterial Activity of PU Foams: Viability Tests

#### 3.4.1. Planktonic Bacteria Cell Count

Antibacterial activity against *E. coli* (ATCC 11229) and *S. aureus* (ATCC 6538) of 1_Irgasan, 1_borneol, 2_terpineol, 4_terpineol, 2_geraniol, 3_geraniol, 1_menthol, and relative control foam devoid of additives (PUC) was evaluated following a AATCC 100-2012 static method, modified on the basis of the specific purposes of this study. Test bacterium culture was grown for 24 h in a suitable nutrient broth (peptone for *E. coli* and Brain Heart Infusion, BHI, for *S. aureus*) and then diluted in Potassium Phosphate Buffer (KPi) at pH 7 for *E. coli* and in KPi/ 20% BHI for *S. aureus* to reach a concentration of 1.5 × 10^5^–3.0 × 10^5^ colony-forming units (CFU) mL^−1^ (inoculum). Three 1 × 1 cm specimens of each functionalized foam were transferred to a vial, 1 mL of diluted solution was added in each vial and incubated at 37 °C for 24 h. After incubation, 1 mL of solution was diluted in KPi, plated in nutrient agar, so that a maximum of 400 CFU was transferred to the plates, incubated for 24 h and the surviving colonies counted.

The bactericidal activity was expressed as the percent viability of the organisms after contact with the test specimen compared to the number of bacterial cells surviving after contact with the control. All tests were performed in triplicate.

#### 3.4.2. Biofilm Viable Cell Counts

Specimens, incubated overnight in 10 mL KPi solution, were gently washed with 3 mL of sterile NaCl 0.9%, and the wastewater was thrown away. They were then immersed in fresh 2 mL sterile NaCl 0.9% and sonicated for 20 min in an ultrasound bath, and 1 mL of the solution was diluted in KPi at 1:100 ratio. A volume of 1 mL of the diluted solution was then plated in nutrient agar incubated for 24 h and the surviving colonies were counted to detect sessile bacteria.

### 3.5. ^1^D ^1^H NMR Characterization of Foam Leaking

Specimens were immersed in 1 mL KPi and kept at 37 °C for 24 h. NMR samples were prepared with this solution by adding 10% D_2_O and trimethylsilyl-2,2,3,3-tetradeuteropropionic acid (TSP) at a final concentration of 0.1 mM.

^1^D ^1^H NMR experiments were recorded at 25 °C on a 500 MHz Bruker Avance II spectrometer. Water suppression was achieved by using excitation sculpting with gradients, with a relaxation delay of 2 s, 128 scans for a total acquisition time of 20 min. Spectra were processed and integrated with Topspin 3.6.2.

## 4. Summary and Conclusions

Several foams have been prepared from a bio-polyol obtained by epoxidized linseed oil and caprylic acid, and TDI in the presence of different monofunctional terpenes such as (+)-menthol, geraniol, terpineol (mixture of isomers), and (+)-borneol. Despite the “green” origin of their ingredients (bio-polyols from vegetable oils), the obtained foams showed good thermal stability combined with good mechanical properties and provided resistance to microbial growth. Menthol-additivated foams showed broad-spectrum antibacterial activity against both Gram+ and Gram−, while those prepared with borneol and terpineol showed selective antibacterial activity against *E. coli* and *S. Aureus*, respectively. NMR analysis indicates that terpene-based foam antibacterial activity is mainly based on a surface contact killing mechanism and it is reasonable to hypothesize that their different bactericidal efficacy is related to the chemical properties of the specific additive and whether it perturbs bacteria cell membranes or targets intracellular sites.

Altogether, the presented findings suggest that the terpene-additivated polyurethane foams, endowed with intrinsic antibacterial activity and devoid of significant active molecule release, represent a step forward towards the development of polyurethane foams for several industrial applications, such as those related to health care, cosmetology, textiles, and food packaging. Indeed, the antimicrobial additive, once incorporated into the polyurethane foam, becomes a permanent part of the structure, conferring antimicrobial action lasting for the effective life of the product. The features of the obtained foams pave the way to very different applications such as in the furniture industry, in which it is crucial to inhibit the proliferation of microbes, reducing microbial-associated odors, and maintain esthetic appeal. Moreover, the formulations proposed could be easily tuned, leading, e.g., to more closed-cell or lower-density structures, thus the antibacterial PU foams could also be used as insulation materials, furthering commercial interest in bio-based products.

The fine tunability of the proposed protocol for foam production, in terms of starting compounds (i.e., polyols from other renewable sources, other classes of bactericidal molecules, etc.) and morphological/mechanical features of the final material, greatly expands the field of application of these new materials.

## Figures and Tables

**Table 1:** Minimal inhibitory concentration (MIC) reported in the literature for the molecules used in this study.

Molecules	MIC (μg/mL)	*E. Coli* Strain	Reference
menthol	<100	ATCC 0157	[41,42]
borneol	512	ATCC 2592	[43]
α-terpineol	725	CMCC (B)44102	[44]
geraniol	5600	ATCC 25922	[45]
Irgasan^®^	0.33	ATCC 11229	[46]

**Table 2:** Properties of bio-polyol ^1^.

Run	Organic Acid	η ^2^ (Pa.s)	*M*_n_ ^3^ (g/mol)	Ð ^3^	Acid Number ^4^ (mg_KOH_/g)	OH Number ^5^ (mg_KOH_/g)	OH Funct. ^6^
BPO	CA	4.70	1769	1.36	3	67	2.1

^1^ Reaction conditions: see [18]. T= 150 °C; CA = caprylic acid. ^2^ Measured at 23 °C. ^3^ Determined by SEC using polystyrene standards. ^4^ Determined according to the IUPAC 2.201 standard method. ^5^ Determined according to ASTM D 4274. ^6^ OH functionality calculated by the formula: *M*_n_ × OH number/(56.1 × 1000).

**Table 3:** Staring formulation of the foam used in this study (PUC).

	PUC
Bio-polyol	44
PPG	56
Glycerol	10.6
Water	1.5
Silicone surfactant	3.9
DABCO	0.6
DBT	0.1
Isocyanate	62
Isocyanante index	100

Units are in parts per hundred parts (pphp).

**Table 4:** Foams with additives used in this study and their core density **^a^**.

Sample	Additive	Additive (wt%)	Density (Kg/m^3^) ^b^
PUC	-	-	120
1_menthol	Menthol	2.3	131
2_terpineol	Terpineol	4.4	116
4_terpineol	Terpineol	8.6	85
2_geraniol	Geraniol	4.4	152
3_geraniol	Geraniol	6.5	131
1_borneol	Borneol	2.3	135
1_irgasan	Irgasan^®^	2.3	86

^a^ The number in the sample name refers to the amount of the additive (example “1” corresponds to 2.3 wt% of additive). ^b^ Determined according to ISO 845:2006; standard deviation up to 10%.

**Table 5:** Mechanical properties of selected foams.

Sample	Young’s Modulus (kPa)	Compression Deflection Value (kPa)
PUC	1181 ± 51	76 ± 6
1_menthol	1100 ± 43	60 ± 4
1_borneol	1305 ± 88	74 ± 6

## Data Availability

Not applicable.
